# Clinical Relevance of Calcifications in Osteosarcoma Lung Metastases: Correlations Among Radiological Patterns, Histological Subtypes, and Chemotherapy Responses

**DOI:** 10.3390/clinpract16090161

**Published:** 2026-08-28

**Authors:** Paolo Spinnato, Mario Simonetti, Maria Carpenzano, Gabriele Bilancia, Nicola Marrone, Annamaria Chiesa, Maddalena Di Carlo, Emanuela Palmerini, Luca Cevolani, Marco Colangeli, Marco Gambarotti, Alberto Righi, Alessandra Longhi

**Affiliations:** 1 Diagnostic and Interventional Radiology, IRCCS Istituto Ortopedico Rizzoli, 40136 Bologna, Italy; 2 Osteoncology, Soft Tissue and Bone Sarcomas, Innovative Therapy Unit, IRCCS Istituto Ortopedico Rizzoli, 40136 Bologna, Italy; 3 Sylvester Comprehensive Cancer Center, University of Miami, Miami, FL 33124, USA; 4 Department of Orthopaedic Oncology, IRCCS Istituto Ortopedico Rizzoli, 40136 Bologna, Italy; 5 Department of Pathology, IRCCS Istituto Ortopedico Rizzoli, 40316 Bologna, Italy

**Keywords:** bone sarcomas, osteosarcoma, lung metastases, disease staging, diagnostic imaging, multidetector computed tomography, sarcoma

## Abstract

**Background/Objectives:** Osteosarcoma is a rare malignancy and the most common primary malignant bone tumor. Metastases commonly affect the lungs and are associated with reduced life expectancy. Calcifications can be present within a metastatic nodule at baseline or can appear during follow-up. We aimed to assess the clinical significance of calcifications in lung metastases, particularly in relation to radiological pattern, histopathological subtype, and treatment response. **Methods:** A retrospective cohort of patients with osteosarcoma and lung metastases at diagnosis was evaluated for the period 2000–2018. The presence of calcifications inside pulmonary lesions on baseline lung CT and/or follow-up was assessed and recorded. Radiological data were then checked against histopathological data (i.e., osteosarcoma subtypes and chemotherapy response with necrosis percentage) and the radiological pattern of the primary tumor (i.e., osteolytic, sclerotic, or mixed pattern). **Results:** Of a cohort of 92 patients, 14 were excluded due to having no baseline or follow-up chest CT. Of the 78 patients with an osteosarcoma diagnosis, lung metastases at diagnosis, and chest CT who were enrolled (42 men and 36 women; mean age: 23.8 years [range, 6–73 years]), 70 (89.7%) had a baseline imaging study of the primary tumor (osteosarcoma). The presence of calcifications in lung metastases at both baseline and follow-up was significantly associated with the radiological pattern of the primary lesion (*p* < 0.0001). Calcifications were not present in the lung metastases of any of the 18 patients (0%) with a lytic radiological pattern at any stage, whereas all 32 patients (100%) with a purely sclerotic pattern had calcifications within lung metastases at baseline or during follow-up; of the 20 patients with a mixed radiological pattern, 11 (55%) had no calcifications and 9 (45.0%) had detectable calcifications. No statistically significant association was found between histopathological subtype and calcification. Osteoblastic osteosarcoma was more frequently associated with calcification (66.0%). During induction chemotherapy, five poor responders and three good responders (necrosis > 90%) developed calcifications (*p* = 0.72; OR = 0.67; 95% CI, 0.10–3.86). **Conclusions:** In patients with osteosarcoma, calcifications within pulmonary metastases are strongly associated with the radiological pattern of the primary tumor, and this association is greatest for a pure sclerotic pattern, followed by the mixed one. We found no statistically significant association between the occurrence of calcifications during follow-up and chemotherapy response measured by necrosis percentage of the surgical specimen in our limited cohort; further research is needed.

## 1. Introduction

Osteosarcoma is the most frequent malignant primary bone tumor. It is known for its highly aggressive behavior, as well as its tendency toward hematogenous spread and lung metastases [1,2]. For this reason, chest CT is still a key diagnostic tool in patients affected by osteosarcoma [3,4,5]. Of all new osteosarcoma cases, 20% to 25% of patients present with synchronous metastases and have a 5-year overall survival of 11% to 40%.

**Figure 1:**
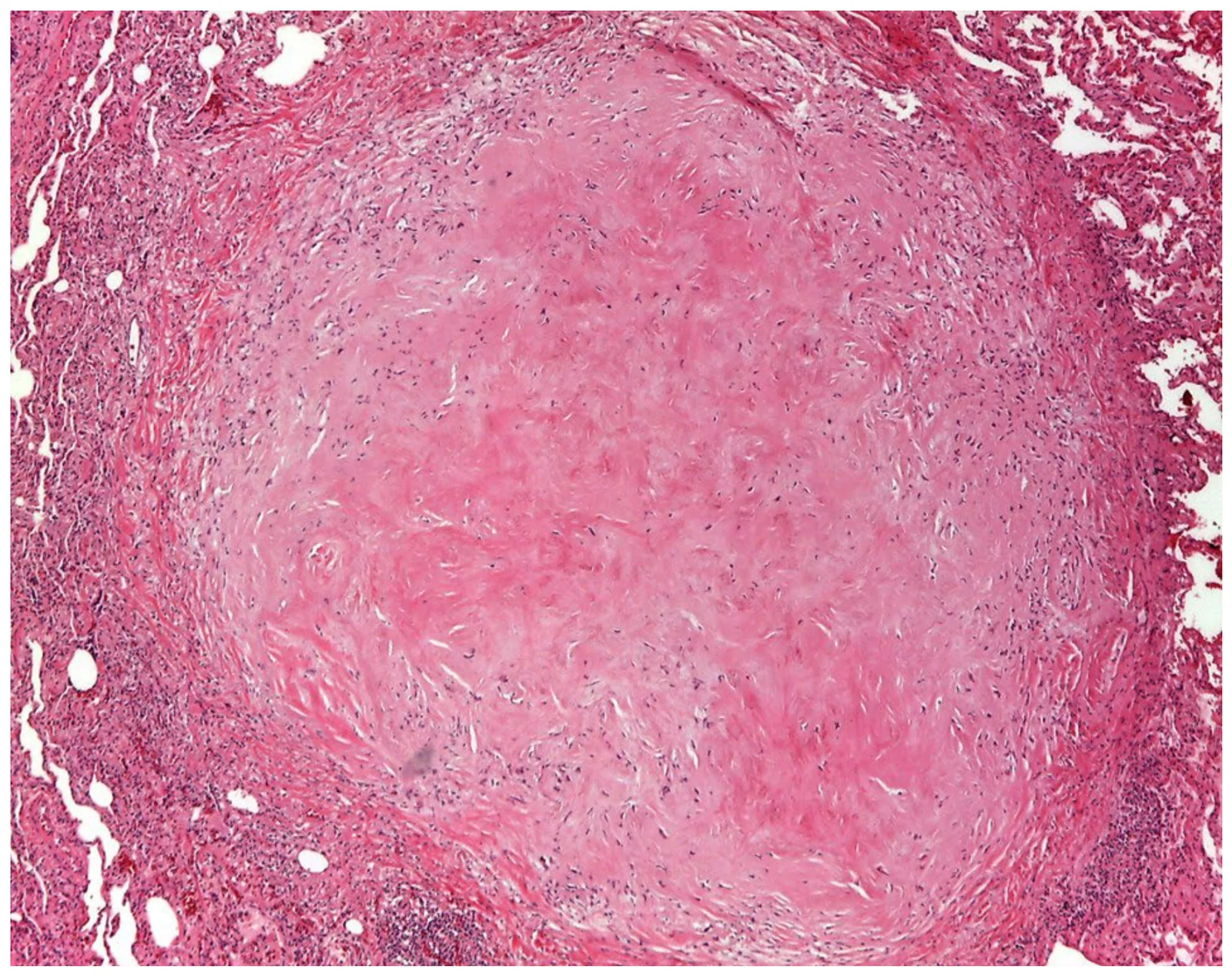
Hematoxylin and eosin staining shows a sclerotic, necrotic, and focally calcified nodule in the lung parenchyma (100× magnification). In a patient with osteosarcoma, this could represent a benign or metastatic etiology and poses a diagnostic challenge.

Primary osteosarcoma is associated with different radiologic patterns [6]. Lung metastases may appear as well-defined nodules with intralesional calcification (or ossification) and/or cavitation [7,8]. However, several benign pulmonary lesions also display internal calcification, such as granulomas (old scars) and hamartomas [8]. This makes the differential diagnosis of calcific nodules challenging in patients with osteosarcoma, particularly at diagnosis. Completely calcified or ossified lung metastasis may even pose significant difficulties in histopathological analysis (Figure 1).

Chemotherapy was introduced as a treatment option for osteosarcoma in 1973, but the most effective options remain unchanged (i.e., methotrexate, cisplatin, and doxorubicin) [3]. Even when a treatment response is observed, solid nodules do not always disappear and morphological changes on chest CT cannot always be relied upon to distinguish necrotic from viable tumor cells, making it difficult to assess treatment response. There are three common non-volumetric variations reported in cohorts during systemic treatments. First, necrosis and cavitation (decrease in density on CT images), which is seen more often in TKI treatment (pazopanib, regorafenib) and can lead to pneumothorax [9,10]. Second, calcification/ossification, which is typical of osteosarcoma metastases and is rare in other solid tumors (both at baseline and during chemotherapy) [8,9]. To the best of our knowledge, there is no scientific evidence supporting that calcifications in lung metastases that develop during chemotherapy are related to treatment response [11]. Third, standardized uptake value reduction in FDG PET-CT studies [10,12,13,14].

The importance of assessing response to chemotherapy in patients who develop lung metastases is critical, especially when surgical treatment is not an option and medical therapy is continued. Ensuring a correct diagnosis (or presumptive diagnosis) of calcific lung nodules at baseline CT is important and should include prognostic information about the possibility of metastatic disease [11,15,16]. Indeed, about 25% of patients with osteosarcoma have indeterminate nodules recorded on baseline chest CT [15].

The aim of our study was to investigate the clinical significance of calcifications in the lung metastases of patients with osteosarcoma by considering the radiological pattern, osteosarcoma histological subtype, and chemotherapy response.

## 2. Materials and Methods

### 2.1. Inclusion Criteria and Patient Population

We retrospectively evaluated patients with a diagnosis of osteosarcoma made at our sarcoma center between 2000 and 2018 if they had lung metastases at diagnosis. All histopathological analyses and diagnoses for primary tumors and lung metastases were performed in our institute’s pathology department by pathologists with more than 15 years of experience diagnosing bone and soft-tissue sarcomas.

Patients were only included if there was a baseline CT thorax at diagnosis and a minimum of 2 years of follow-up imaging. Lung metastases were diagnosed by imaging (chest CT and PET-CT) and confirmed by a multidisciplinary sarcoma tumor board, both at baseline and at any follow-up. The lung nodule morphology, including dimensions and changes in size during follow-up, was considered when diagnosing metastatic lung disease.

### 2.2. Radiological Analyses

Baseline imaging of the primary tumor was evaluated by conventional radiography and CT, and the osteosarcoma was classified as sclerotic, lytic, or mixed.

Available chest CTs were evaluated for the presence or absence of calcifications in pulmonary lesions at baseline and/or the appearance of calcifications during follow-up. All pulmonary nodules were evaluated. Evaluation of chest CTs was performed blindly, without access to relevant clinical or histopathological data, using lung, soft-tissue, and bone windows. Pulmonary metastases were evaluated by semi-quantitative visual assessment, with patients subdivided into three groups: (i) metastatic with calcifications from baseline (in at least one metastasis), (ii) metastatic with calcifications appearing at follow-up (in at least one metastasis), and (iii) metastatic without any calcifications.

Two expert oncological radiologists (P.S. and M.S.) evaluated the images (16 years’ and 5 years’ experience, respectively), discussing cases until reaching a consensus if needed. All radiological images and reports were assessed on a Picture Archiving and Communication System (PACS-Carestream Vue PACS v. 11.4.1.1102, Philips Healthcare, Amstelplein 2, 1096 BC Amsterdam, The Netherlands).

### 2.3. Clinical Analyses

An expert musculoskeletal oncologist (A.L.) with 30 years of experience evaluated clinical data from medical records. The histopathological diagnosis of primary osteosarcoma was based on surgical biopsy, with patients divided by histological subtype. If the primary tumor was resected, by either amputation or limb-sparing surgery, the percentage necrosis in the whole specimen was assessed by histopathology to evaluate and classify the response to chemotherapy. A 90% prognostic cut-off was then used to classify good responders (necrosis ≥ 90%) and poor responders (necrosis < 90%) [17].

### 2.4. Statistical Analysis

To assess whether the development of calcifications in pulmonary metastases during follow-up was associated with the presence of necrosis, patients were classified as good responders or poor responders based on the 90% necrosis threshold [17]. A 2 × 2 contingency table comparing patients with non-calcified metastases at diagnosis that subsequently calcified versus all other patients was analyzed using Fisher’s exact test.

To further explore whether pulmonary metastasis calcification at diagnosis and at a later time was associated with specific histological subtypes, tumors were dichotomized into osteoblastic and non-osteoblastic osteosarcoma. A 2 × 2 contingency table was constructed and analyzed using Fisher’s exact test.

The association between the radiological pattern of the primary tumor and the calcification pattern of lung metastases was assessed using Fisher’s exact test, implemented with Monte Carlo simulation to account for the presence of zero cells and small subgroup sizes in a 3 × 3 contingency table. Effect size was quantified using Cramér’s V with 95% confidence intervals.

All analyses were performed in RStudio (2026.01.1 Build 403).

### 2.5. Ethics

This retrospective study was conducted in line with the criteria set by the Declaration of Helsinki and was approved by the local Institutional Review Board.

## 3. Results

### 3.1. Metastatic Osteosarcoma Patients’ Cohort

**Figure 2:**
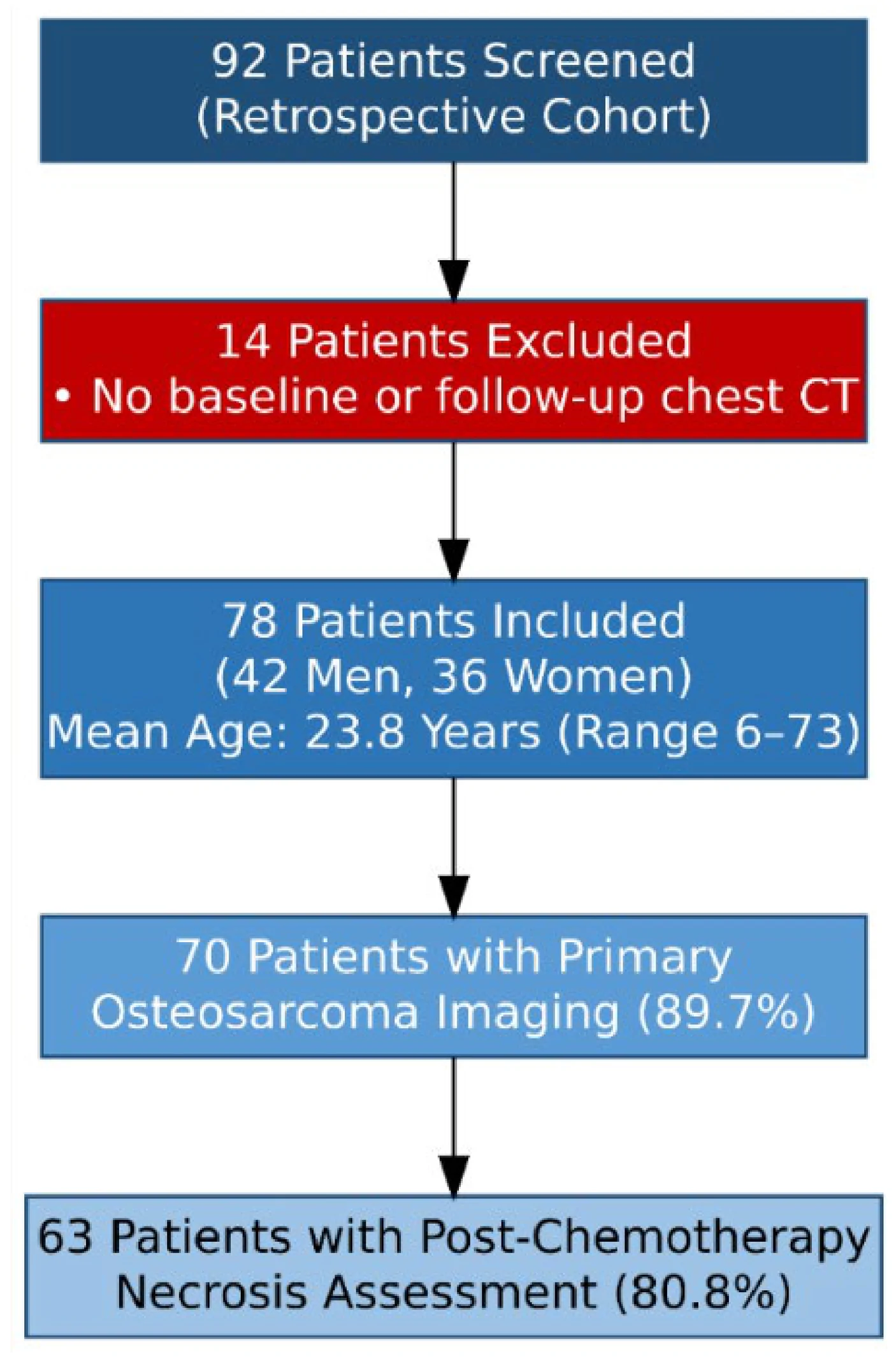
Flow-chart of patient selection.

Of the 92 eligible patients, 14 were excluded due to missing chest CT at baseline or during follow-up. The final cohort included 78 patients (42 men; 36 women) aged 6 to 73 years (mean, 23.8 years). Patient selection is summarized in Figure 2.

### 3.2. Association Between Primary Osteosarcoma Radiological Patterns and Lung Metastasis Calcification

We excluded eight patients who had no baseline imaging of the primary tumor, leaving 70 patients (89.7%) with adequate CT and/or conventional radiography of the primary lesion to allow for the assessment of the main radiological pattern: 32 patients presented with a purely sclerotic radiological pattern (45.7%), 20 with a mixed pattern (28.6%), and 18 with a purely lytic pattern (25.7%).

**Figure 3:**
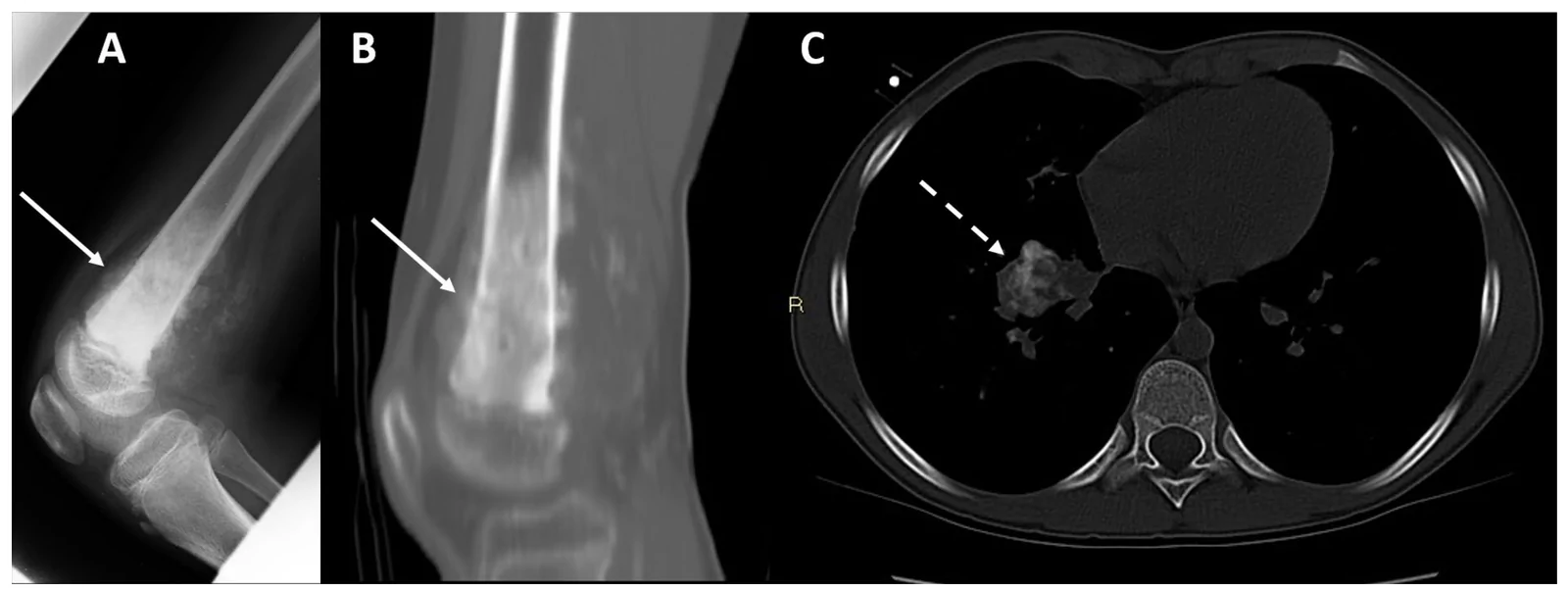
An 11-year-old boy with high-grade osteoblastic osteosarcoma of the right distal femur. Panels (**A**) (conventional radiology) and (**B**) (CT with sagittal reconstruction) show a purely sclerotic radiologic pattern; Panel (**C**) (baseline CT with bone window) shows lung metastases with diffuse calcifications. Lesions are indicated by arrows.

**Figure 4:**
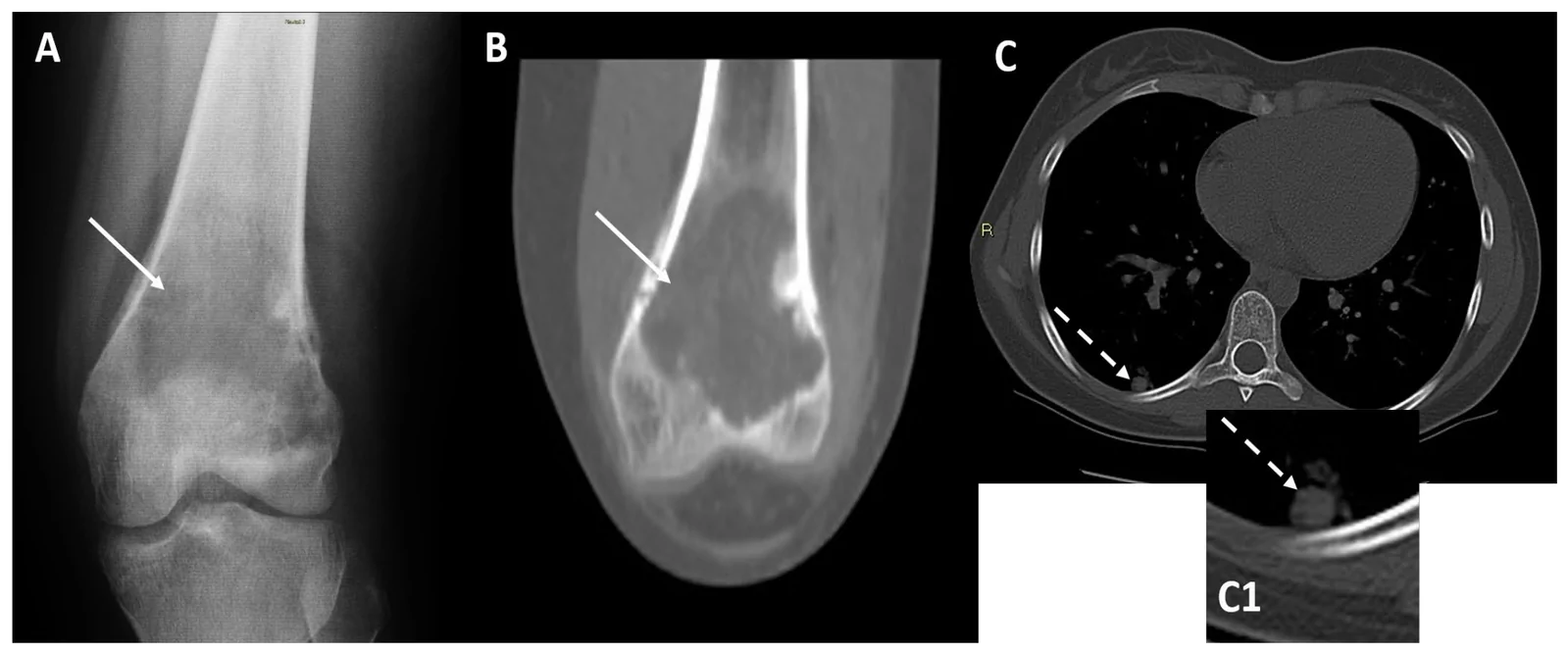
A 16-year-old girl affected by telangiectatic osteosarcoma of the left distal femur. Panel (**A**) (conventional radiology) and Panel (**B**) (CT with coronal reconstruction) show a purely lytic radiologic patter-; Panel (**C**) (Baseline CT with bone window; magnified in (**C1**)) shows lung metastases without internal calcification. Lesions are indicated by arrows.

All 32 patients with a purely sclerotic pattern had calcifications in lung metastases, with calcification present on baseline CT in 26 cases (81.2%) and developing during follow-up in six cases (18.8%) (Figure 3). Of the 20 patients with a mixed radiological pattern, 11 (55.0%) had no calcifications, and nine (45.0%) had detectable calcifications (three at baseline and six at follow-up). None of the 18 patients with a purely lytic radiological pattern had calcifications within lung metastases at + baseline or during follow-up (Figure 4).

**Figure 5:**
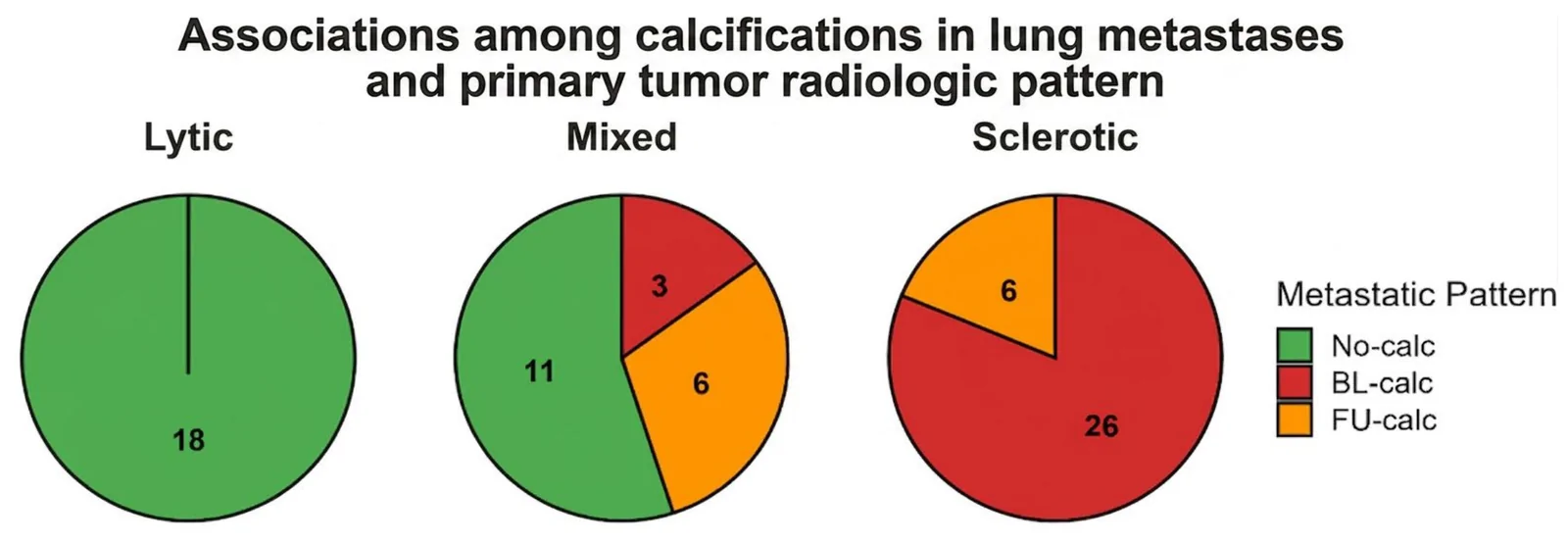
Pie chart of the associations between calcifications (presence, absence, and appearance) and the primary tumor’s radiologic patterns among the 70 patients included in this specific analysis. From left to right, the charts correspond to lytic, mixed, and sclerotic radiologic patterns. Each slice represents a metastatic pattern: Green = No-calc (lung metastases without calcification at baseline or follow-up); orange = FU-calc (lung metastases with calcification appearing during follow-up; red = BL-calc (lung metastases with calcifications present since baseline).

The association between lung metastasis calcification and the radiological pattern of the primary tumor was statistically significant according to Fisher’s exact test (Monte Carlo simulation, *p* < 0.0001), as shown in Table 1. The effect size was large, as indicated by a Cramér’s V of 0.64 (95% CI, 0.45–0.79), confirming a strong association between the two variables (represented graphically in Figure 5).

### 3.3. Association Between Osteosarcoma Histological Subtypes and Lung Metastasis Calcification

Osteosarcoma distribution by histopathological subtype is shown for the 78 patients in Table 2, with comparison by the presence or absence of calcifications in lung metastases.

**Figure 6:**
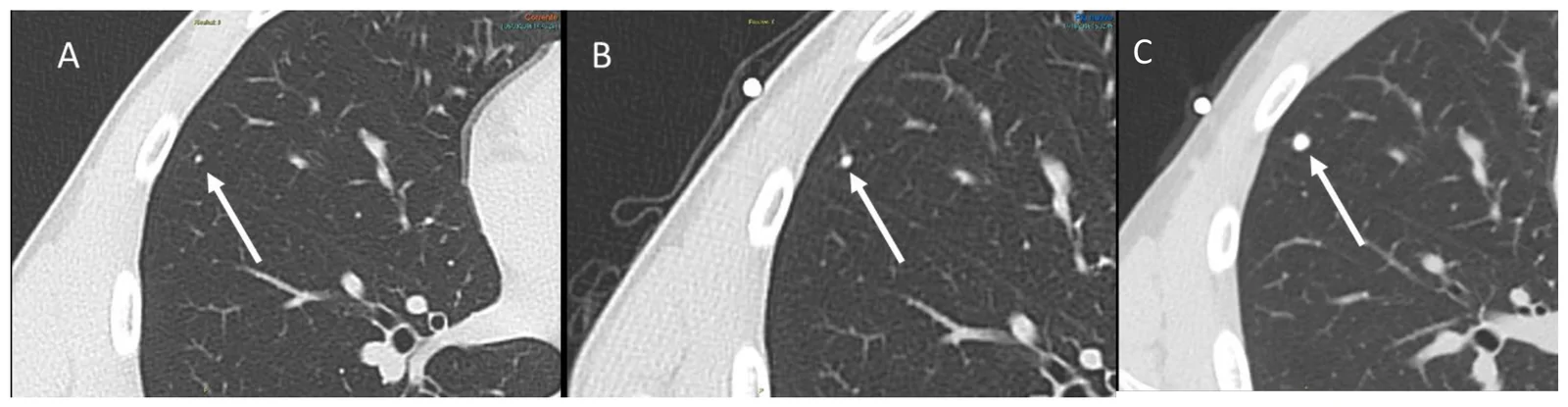
Chest CTs of a 15-year-old male affected by high-grade (Broders’ grade 4) osteoblastic osteosarcoma. Calcified lung metastasis is shown that progressively increased in size (arrows). Panel (**A**) = baseline; Panel (**B**) = 1st follow-up; Panel (**C**) = 2nd follow-up.

Assessment of lung metastasis calcification by osteosarcoma subtype revealed no statistically significant results:

- Twenty-five patients (50.0%) with the osteoblastic subtype showed calcified lung metastases at diagnosis;
- Seventeen patients (34.0%) with the osteoblastic subtype showed non-calcified lung metastases at diagnosis that remained non-calcified during chemotherapy (Figure 6);
- Eight patients (16.0%) with the osteoblastic subtype showed non-calcified lung metastases at diagnosis that subsequently developed calcifications during chemotherapy;
- Two patients (50.0%) with the chondroblastic subtype showed calcified lung metastases at diagnosis;
- Two patients (50.0%) with the chondroblastic subtype showed non-calcified lung metastases at diagnosis that remained non-calcified during chemotherapy;
- One patient (33.3%) with the fibroblastic subtype showed calcified lung metastases at diagnosis;
- Two patients (66.7%) with the fibroblastic subtype showed non-calcified lung metastases at diagnosis that remained non-calcified during chemotherapy;
- Four patients (44.4%) with the osteoblastic–chondroblastic subtype showed calcified lung metastases at diagnosis;
- Four patients (44.4%) with the osteoblastic–chondroblastic subtype showed non-calcified lung metastases at diagnosis that remained non-calcified during chemotherapy;
- One patient (11.1%) with the osteoblastic–chondroblastic subtype showed non-calcified lung metastases at diagnosis that developed calcifications during chemotherapy;
- Three patients (75.0%) with the osteoblastic–fibroblastic subtype showed non-calcified lung metastases at diagnosis that remained non-calcified during chemotherapy;
- One patient (25.0%) with the osteoblastic–fibroblastic subtype showed non-calcified lung metastases at diagnosis that developed calcifications during chemotherapy;
- All three patients (100%) with the spindle cell subtype showed non-calcified lung metastases at diagnosis that remained non-calcified during chemotherapy;
- The only patient (100%) with the osteoblastic–telangiectatic subtype showed non-calcified lung metastases at diagnosis that remained non-calcified during chemotherapy;
- The only patient (100%) with the telangiectatic subtype showed non-calcified lung metastases at diagnosis that remained non-calcified during chemotherapy;
- The only patient (100%) with the dedifferentiated parosteal subtype showed calcified lung metastases at diagnosis;
- The only patient (100%) with the chondroblastoma-like subtype developed calcifications during chemotherapy; and
- The only patient (100%) with the small cell chondroblastic subtype developed calcifications during chemotherapy.

Even when comparing the higher percentage of calcified metastases that appeared at baseline or at later time in osteoblastic subtypes versus other subtypes, Fisher’s exact test revealed no statistically significant association between subtype and calcification at diagnosis (*p* = 0.06; OR = 0.39; 95% CI: 0.15–1.01). Table 3 summarizes the comparison between classic/osteoblastic osteosarcoma versus other histological subtypes.

### 3.4. Association Between Chemotherapy Treatment Response and Development of Lung Metastasis Calcification

When restricting the analysis to patients with data on both imaging and histological response (*n* = 63), Fisher’s exact test did not show a statistically significant association between delayed calcification and good histological response (*p* = 0.72; OR = 0.67; 95% CI: 0.10–3.86; Table 4).

## 4. Discussion

Our study reveals, for the first time, that the presence or absence of calcifications in lung metastases at baseline and follow-up is significantly associated with the radiological pattern of the primary osteosarcoma. Specifically, we showed that lytic lesions are almost exclusively linked to non-calcified metastases, sclerotic lesions are predominantly associated with baseline-calcified metastases, and mixed lesions show an intermediate distribution.

We found no significant correlation between the osteosarcoma histotype and calcification in lung metastases that might help predict the disease course. The observational data in this study suggest a nearly equal distribution of calcified and non-calcified metastases across the osteoblastic, chondroblastic, and osteochondroblastic subtypes. In the fibroblastic subtype, there was a slight increase in non-calcified lesions versus calcified lesions (2:1 ratio). However, the number of patients was too small, especially in the rarer histological subgroups, so the result was not statistically significant.

In this small exploratory analysis using a heterogeneous comparator group, no statistically significant association was detected between delayed pulmonary metastasis calcification and primary tumor histological response. Indeed, in our cohort, we observed a small number of patients who developed calcifications during chemotherapy (three good responders, five poor responders), although the sample size is too limited to draw firm conclusions about any association. A larger series will be needed to verify these data, which is of particular importance because research has yet to clarify the relevance of calcification that appears in lung metastases during chemotherapy for osteosarcoma [18]. Some unknown factors could play a role in the development of calcifications. Future studies should include data from serial FDG PET-CT scans to determine whether calcification provides evidence of a chemotherapy response. Previous studies have reported a moderate association between increasing mineralization of the primary osteosarcoma and histological response. Whether calcification developing in pulmonary metastases reflects a different biological process remains uncertain and was not directly evaluated in the present study [19,20]. On the other hand, at diagnosis, calcifications are considered the most reliable radiological indicator for confirming lung metastases in patients with osteosarcoma [21,22,23,24,25,26].

This original research has some limitations. First, its retrospective nature is subject to bias and unreported or incomplete data. Several of the 78 patients in the cohort had missing data: eight had no recorded radiological study of the primary tumor, and 15 had no recorded assessment of necrosis. Second, the radiological analysis was performed by consensus only, and we did not assess interobserver and intraobserver agreement. Third, the radiological analyses performed both on the primary tumor (i.e., lytic, sclerotic, mixed) and on the lung metastases (i.e., presence/absence of calcification) did not include complex quantitative analyses. Notably, for chemotherapy response assessment, we only compared patients with calcification appearance versus all others (never calcified or already calcified). This reduces the precision of our results but does not detract from their utility as a proof of concept that should stimulate further research and clinical debate. Fourth, our research does not include an analysis of the associations between lung metastasis calcification and survival (overall and sarcoma-specific survival), which could be addressed in future research. Also, metastatic disease was diagnosed by CT and sarcoma tumor board discussion, without histological analysis. Fifth, in this small exploratory analysis using a heterogeneous comparator group, no statistically significant association was detected between delayed pulmonary metastasis calcification and primary tumor histological response. Finally, we could not conduct a multivariate analysis because data were missing for several variables and only the radiological pattern was statistically significant in the univariate analysis.

## 5. Conclusions

This study revealed a strong and significant association between the radiological patterns of osteosarcoma and the presence of calcifications within lung metastases:

- Lytic lesions were almost exclusively associated with non-calcified lung metastases.
- Sclerotic lesions were strongly associated with calcified metastases at baseline or during follow-up.
- Mixed lesions showed an intermediate behavior in terms of lung metastasis calcification.

We conclude that the intrinsic nature of osteosarcoma, as reflected by its radiological pattern, is strongly correlated with the resulting pattern of calcifications in lung metastases.

## Figures and Tables

**Table 1:** Contingency table showing the radiologic pattern distributions of the primary tumor (lytic, mixed or sclerotic) and the metastatic pattern categories in 70 patients. There is a strong association. Patterns of calcification: 0 = None (lung metastases without calcification at baseline or follow-up); 1 = During follow-up (FU) (lung metastases with calcifications that appeared during follow-up); 2 = From baseline (lung metastases with calcifications present from baseline).

Radiologic Pattern	Metastatic Pattern of Calcification			Total
	0 = None	1 = During FU	2 = From Baseline	
Lytic	18	0	0	18
Mixed	11	6	3	20
Sclerotic	0	6	26	32
Total	29	12	29	70

**Table 2:** Radiological pattern of primary osteosarcoma and lung metastasis calcification, reported as number of patients (% among the selected subtype).

Histological Subtype	Calcified Lung Metastases		Non-Calcified Lung Metastases *	Total
	Present at Baseline	Appearing at Follow-Up		
Osteoblastic–telangiectatic	0 (0%)	0 (0%)	1 (100%)	1
Spindle cell	0 (0%)	0 (0%)	3 (100%)	3
Chondroblastic	2 (50.0%)	0 (0%)	2 (50.0%)	4
Chondroblastoma-like	0 (0%)	1 (100%)	0 (0%)	1
Fibroblastic	1 (33.3%)	0 (0%)	2 (66.7%)	3
Osteoblastic	25 (50.0%)	8 (16.0%)	17 (34.0%)	50
Osteoblastic–chondroblastic	4 (44.4%)	1 (11.1%)	4 (44.4%)	9
Osteoblastic–fibroblastic	0 (0%)	1 (25.0%)	3 (75.0%)	4
Dedifferentiated parosteal	1 (100%)	0 (0%)	0 (0%)	1
Small cell chondroblastic	0 (0%)	1 (100%)	0 (0%)	1
Telangiectatic	0 (0%)	0 (0%)	1 (100%)	1
Total	33	12	33	78

* Metastases without calcifications (at baseline and during follow-up CTs).

**Table 3:** Association between histological subtype (osteoblastic vs. non-osteoblastic) and presence of calcified pulmonary metastases at diagnosis at any time.

Histological Group	Calcified	Non-Calcified	Total
Osteoblastic	33	17	50
Non-osteoblastic (others)	12	16	28
Total	45	33	78

**Table 4:** Distribution of treatment response by pulmonary metastasis calcification pattern. The other group included patients with no calcification development or with calcification present from baseline.

Calcification Pattern	Good Responders	Poor Responders	Total
Developed during chemotherapy	3	5	8
Other	26	29	55
Total	29	34	63

## Data Availability

The original contributions presented in this study are included in the article. Further inquiries can be directed to the corresponding author. Further clinical data on this patient cohort can be found in a published scientific abstract [27].

## Abbreviations

The following abbreviations are used in this manuscript:

CT	Computed Tomography
FDG	Fluorodeoxyglucose
OR	Odds ratio
PET	Positron Emission Tomography

## References

[1] Kundu Z.S. (2014). Classification, imaging, biopsy and staging of osteosarcoma. Indian J. Orthop..

[2] Nguyen J.C., Baghdadi S., Pogoriler J., Guariento A., Rajapakse C.S., Arkader A. (2022). Pediatric Osteosarcoma: Correlation of Imaging Findings with Histopathologic Features, Treatment, and Outcome. Radiographics.

[3] Elliott L., Loeb D.M., Trucco M. (2026). Management of pediatric bone sarcomas. Curr. Opin. Pediatr..

[4] Adriaansen L.M.E., Merks J.H.M., van Dalen E.C., Andersen K.F., Asaftei S.D., Bernabeu D., Boye K., Campello A., Capra M., Costa Dias S. (2026). European guideline for imaging of primary paediatric and adult osteosarcoma and Ewing sarcoma: Systematic review and joint statement by the FOSTER consortium, Euro Ewing Consortium, European Society of Paediatric Radiology, and the European Association of Nuclear Medicine. Lancet Oncol..

[5] Duczkowski M., Duczkowska A., Olwert A., Michalak E., Bilska K., Klepacka T., Rychłowska-Pruszyńska M., Raciborska A., Bekiesińska-Figatowska M. (2024). Predictors of pulmonary metastases on chest computed tomography in children and adolescents with osteosarcoma-tips for qualifying patients for thoracotomy. BMC Pediatr..

[6] Crombé A., Simonetti M., Longhi A., Hauger O., Fadli D., Spinnato P. (2024). Imaging of Osteosarcoma: Presenting Findings, Metastatic Patterns, and Features Related to Prognosis. J. Clin. Med..

[7] Ciccarese F., Bazzocchi A., Ciminari R., Righi A., Rocca M., Rimondi E., Picci P., Bacchi Reggiani M.L., Albisinni U., Zompatori M. (2015). The many faces of pulmonary metastases of osteosarcoma: Retrospective study on 283 lesions submitted to surgery. Eur. J. Radiol..

[8] Spinnato P. (2024). Calcified Osteosarcoma Lung Metastases. Radiology.

[9] Briccoli A., Rocca M., Salone M.C., Di Fiore M., Vanel D., Balladelli A., Alberghini M. (2009). “Bubble-like” lung metastases in osteosarcoma patients. Eur. J. Radiol..

[10] Cicchetti G., Marano R., Strappa C., Amodeo S., Grimaldi A., Iaccarino L., Scrocca F., Nardini L., Ceccherini A., Del Ciello A. (2025). New insights into imaging of pulmonary metastases from extra-thoracic neoplasms. Radiol. Med..

[11] Chiesa A.M., Spinnato P., Miceli M., Facchini G. (2021). Radiologic Assessment of Osteosarcoma Lung Metastases: State of the Art and Recent Advances. Cells.

[12] Davis J.C., Daw N.C., Navid F., Billups C.A., Wu J., Bahrami A., Jenkins J.J., Snyder S.E., Reddick W.E., Santana V.M. (2018). 18F-FDG Uptake During Early Adjuvant Chemotherapy Predicts Histologic Response in Pediatric and Young Adult Patients with Osteosarcoma. J. Nucl. Med..

[13] Im H.J., Zhang Y., Wu H., Wu J., Daw N.C., Navid F., Shulkin B.L., Cho S.Y. (2018). Prognostic Value of Metabolic and Volumetric Parameters of FDG PET in Pediatric Osteosarcoma: A Hypothesis-generating Study. Radiology.

[14] Dutour A., Decouvelaere A.V., Monteil J., Duclos M.E., Roualdes O., Rousseau R., Marec-Bérard P. (2009). 18F-FDG PET SUVmax correlates with osteosarcoma histologic response to neoadjuvant chemotherapy: Preclinical evaluation in an orthotopic rat model. J. Nucl. Med..

[15] Ghosh K.M., Lee L.H., Beckingsale T.B., Gerrand C.H., Rankin K.S. (2018). Indeterminate nodules in osteosarcoma: What’s the follow-up?. Br. J. Cancer.

[16] Brader P., Abramson S.J., Price A.P., Ishill N.M., Emily Z.C., Moskowitz C.S., La Quaglia M.P., Ginsberg M.S. (2011). Do characteristics of pulmonary nodules on computed tomography in children with known osteosarcoma help distinguish whether the nodules are malignant or benign?. J. Pediatr. Surg..

[17] Bacci G., Mercuri M., Longhi A., Ferrari S., Bertoni F., Versari M., Picci P. (2005). Grade of chemotherapy-induced necrosis as a predictor of local and systemic control in 881 patients with non-metastatic osteosarcoma of the extremities treated with neoadjuvant chemotherapy in a single institution. Eur. J. Cancer.

[18] Gassiamis A., Tsakonas G., Soukouli G., Mylonakis N., Karabelis A., Kosmas C. (2006). Diffuse calcification of metastases after intensive multiagent chemotherapy in widespread osteosarcoma leading to death in a 18-year-old male: Report of a case and literature review. Med. Oncol..

[19] Henderson E.R., Xu X., Pogue B.W., Samkoe K.S., Anderson M.E. (2020). Osteosarcoma mineralization changes on radiographs have moderate correlation to chemotherapy response using bone subtraction methodology. Ann. Jt..

[20] Smith J., Heelan R.T., Huvos A.G., Caparros B., Rosen G., Urmacher C., Caravelli J.F. (1982). Radiographic changes in primary osteogenic sarcoma following intensive chemotherapy. Radiological-pathological correlation in 63 patients. Radiology.

[21] Martínez Sánchez H., Cañete Nieto A., Sánchez Mateos D., Benavent N., Escrivá Fernández J., Sánchez Robles A., Galley Martín C.P., García Vázquez J., Salom Taverner M., Marco Macián A. (2025). Lung nodules in pediatric osteosarcoma: Calcification as the most reliable radiological indicator to confirm metastasis. Pediatr. Radiol..

[22] Silva J.A.M., Marchiori E., Amorim V.B., Barreto M.M. (2023). CT features of osteosarcoma lung metastasis: A retrospective study of 127 patients. J. Bras. Pneumol..

[23] Khan A.N., Al-Jahdali H.H., Allen C.M., Irion K.L., Al Ghanem S., Koteyar S.S. (2010). The calcified lung nodule: What does it mean?. Ann. Thorac. Med..

[24] Rastogi R., Garg R., Thulkar S., Bakhshi S., Gupta A. (2008). Unusual thoracic CT manifestations of osteosarcoma: Review of 16 cases. Pediatr. Radiol..

[25] Li J., Deng C., Yuan J., Jia H., Peng L. (2024). Association of CT characteristics of osteosarcoma lung metastases with spontaneous pneumothorax: A retrospective analysis. J. Thorac. Dis..

[26] Kusma J., Young C., Yin H., Stanek J.R., Yeager N., Aldrink J.H. (2017). Pulmonary Nodule Size < 5 mm Still Warrants Investigation in Patients with Osteosarcoma and Ewing Sarcoma. J. Pediatr. Hematol. Oncol..

[27] Longhi A., Broll V., Righi A., Carella A., Pierini M., Ferrari C., Cesari M., Hakim R., Paioli A., Palmerini E. (2021). Metastatic osteosarcoma at diagnosis: Analysis of 92 cases from a single institution. J. Clin. Oncol..

